# Adaptive auditory assistance for stride length cadence modification in older adults and people with Parkinson’s

**DOI:** 10.3389/fphys.2024.1284236

**Published:** 2024-02-07

**Authors:** Tina L. Y. Wu, Anna Murphy, Chao Chen, Dana Kulić

**Affiliations:** ^1^ Faculty of Engineering, Monash University, Melbourne, VIC, Australia; ^2^ Clinical Research Centre for Movement Disorders and Gait, Monash Health, Clayton, VIC, Australia

**Keywords:** adaptive auditory cueing, rhythmic auditory stimulation, gait rehabilitation, Parkinson’s disease, aging gait

## Abstract

Gait rehabilitation using auditory cues can help older adults and people with Parkinson’s improve walking performance. While auditory cues are convenient and can reliably modify gait cadence, it is not clear if auditory cues can reliably modify stride length (SL), another key gait performance metric. Existing algorithms also do not address habituation or fluctuation in motor capability, and have not been evaluated with target populations or under dual-task conditions. In this study, we develop an adaptive auditory cueing framework that aims to modulate SL and cadence. The framework monitors the gait parameters and learns a personalized cue-response model to relate the gait parameters to the input cues. The cue-response model is represented using a multi-output Gaussian Process (MOGP) and is used during optimization to select the cue to provide. The adaptive cueing approach is benchmarked against the fixed approach, where cues are provided at a fixed cadence. The two approaches are tested under single and dual-task conditions with 13 older adults (OA) and 8 people with Parkinson’s (PwP). The results show that more than half of the OA and PwP in the study can change both SL and cadence using auditory cues. The fixed approach is best at changing people’s gait without secondary task, however, the addition of the secondary task significantly degrades effectiveness at changing SL. The adaptive approach can maintain the same level of SL change regardless of the presence of the secondary task. A separate analysis is conducted to identify factors that influence the performance of the adaptive framework. Gait information from the previous time step, along with the previous input cue, can improve its prediction accuracy. More diversity in the initialization data can also improve the GP model. Finally, we did not find a strong correlation between stride length and cadence when the parameters are contingent upon input cues.

## 1 Introduction

Gait rehabilitation is an important physical therapy that helps preserve or improve walking performance and counteract the symptoms of neurological disorders and aging. Gait rehabilitation can be administered through rhythmic auditory stimulation (RAS). The stimulation utilizes a phenomenon known as entrainment (Thaut et al., 2015), the coupling of a sensory system with the motor system through the use of auditory cues, such as using metronome beats or music with a strong rhythm (Thaut et al., 2015).

RAS is an attractive solution to gait rehabilitation due to its low cost and ease of use even in unsupervised home training (Ghai et al., 2018a). For neurological disorders like Parkinson’s Disease (PD), the symptoms of gait impairment can become resistant to pharmaceutical treatments over time (Cilia et al., 2015) and therefore physical therapy is essential to the treatment paradigm. RAS is commonly evaluated using spatial-temporal gait parameters, such as cadence, stride length, and gait speed. Previous literature has shown that RAS can influence cadence in both older adults (OA) and people with Parkinson’s (PwP) (Ghai et al., 2018b; Ghai et al., 2018a). However, the effect of RAS on stride length can be mixed. While systematic reviews indicate auditory cues can positively influence stride length (e.g., (Ghai et al., 2018b; Ghai et al., 2018a; Spaulding et al., 2013)), individual experiments have suggested the cues either have no effect on stride length or the effect depends on the pace of the cues (i.e., cues faster than baseline can negatively impact stride length) (Suteerawattananon et al., 2004; Willems et al., 2006; del Olmo and Cudeiro, 2005).

In addition to the mixed results of RAS on stride length, there are other challenges associated with existing approaches to cue generation, which typically provide cues at a fixed frequency (Ginis et al., 2018; Sweeney et al., 2019). The lack of cue adaptation means the approach cannot handle habituation and change in motor performance (Ginis et al., 2018; Sweeney et al., 2019). Habituation can affect both OA and PwP, as cues at a fixed pace can become less salient over long-term use. People’s motor performance can also change due to age or fluctuate due to medication cycle, where the same static cue may not be as effective. Recently, researchers have proposed adaptive cue generation to address these challenges, e.g., (Zhang et al., 2022; Zhang et al., 2020). However, studies to date are mostly conducted with healthy adults instead of the target populations. In addition, these studies often focus on using RAS to increase gait speed without considering the coupling between stride length and cadence, known as the stride length cadence relationship (SLCrel). Different individuals may exhibit different SLCrels; common SLCrels include positive linear, negative quadratic, and negative linear relationships (Egerton et al., 2011). Gait impairment in PD, known as freezing of gait, is often preceded by a breakdown of the normal SLCrel, where an increase in cadence is followed by an abnormal decrease in stride length (Sweeney et al., 2019). RAS that only consider gait speed can potentially be detrimental in PD, as a faster gait speed can be achieved by increasing cadence without increasing stride length, which may lead to freezing. Finally, adaptive RAS has not been studied under the dual-task scenario, where participants need to walk while performing a secondary task. The capacity to perform a secondary task during gait is essential for daily activities and can be impaired due to age or disease, which can increase the risk of falls (O’Shea et al., 2002; Beurskens and Bocks, 2012).

There are two aims in this exploratory study. The first aim is to compare the performance of the adaptive cue provision framework first proposed in (Wu et al., 2023b) to the state-of-the-art fixed cue approach. Toward the first aim, we conduct an exploratory study with OA and PwP under two task conditions: with and without secondary task. The second aim is to explore how the adaptive framework’s performance is impacted by the experiment procedure and the model parameters. The performance of the adaptive framework is evaluated after the first 12 participants (11 OA and 1 PwP) and changes to the model parameters and study procedure are instituted for the remaining participants (1 OA and 7 PwP). Based on the two aims, the contribution of the study is two-fold. First, we study the effect of RAS with respect to the cueing approaches, participant group, and task conditions, using a representative population. Overall, the OA and PwP respond to the fixed/adaptive RAS in a similar capacity. Adding a secondary task decreases gait performance in both groups. The fixed approach is best at changing stride length in the condition without secondary task. The adaptive approach can encourage the same level of stride length change regardless of the presence of a secondary task. The second contribution identifies key factors that improve adaptive framework performance, which includes augmenting the input with information from the previous gait state and adding more diversity to the initialization data.

## 2 Methods

### 2.1 Proposed approach

**FIGURE 1:**
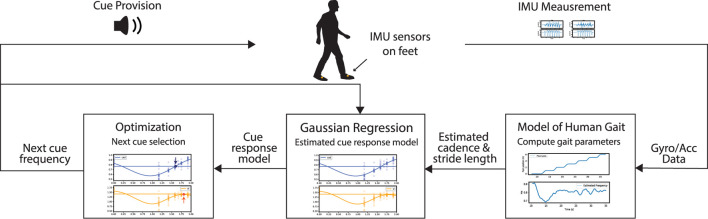
The adaptive framework consists of three main functionalities: estimating gait performance, modeling cue influence on gait, and selecting new cues.

The proposed framework adapted from (Wu et al., 2023b) is summarized in the following section and illustrated in Figure 1. The framework has three primary functions: estimate gait parameters, model cue influence on gait, and select the next cue. We use a Gaussian Process (GP) to relate the input cue and the resultant gait parameters.

#### 2.1.1 Estimate gait parameters

To estimate stride length and cadence in an unconstrained walking environment, an IMU-based (inertial measurement unit) algorithm is implemented, where the sensor is fixed onto the foot. To estimate stride length, the Madgwick filter (Madgwick et al., 2010) is first used to estimate IMU orientation. The accelerometer data from the IMU is then transformed to the world coordinate, as the stride length is equal to the horizontal distance traveled in the world coordinate. Prior to integrating the accelerometer signals, the zero velocity update (ZVU) method is applied to correct gyroscope drift during the stance phase of the gait cycle (Skog et al., 2010). ZVU requires distinguishing between the swing and stance phases, which is based on the modified version of (Van Nguyen and La, 2016) described in our previous work (Wu et al., 2023b) to improve robustness across participants. The gait estimation algorithm has an estimation error and standard deviation of −0.09 ± 0.03 m during straight-line walking and the error for circle-walking is −0.024 ± 0.19 m compared to measurements obtained using Vicon motion capture.

#### 2.1.2 Model cue-influence on gait

A sparse multi-output Gaussian Process (MOGP) is used to model the gait parameters (i.e., stride length and cadence) as a function of the input(s). The model is as follows:

$$\begin{array}{ll} Y & =f\left(x\right)+\epsilon =Wg\left(x\right)+\epsilon ,\;\text{where} \end{array}$$ (1)

$$\begin{array}{ll} g\left(x\right) & ={\left\{g_{q}\left(x\right)\right\}}_{q=1}^{Q},\;g_{q}\left(\cdot \right)∼GP\left(0,k_{q}\left(\cdot ,\cdot^{\prime }\right)\right)\\ \epsilon & =N\left(0,\;\sigma^{2}\right) \end{array}$$ (2)

where **x** is the input. **g**(**x**) is a collection of Q independent latent GPs. The outputs, **Y**, are assumed to be linearly correlated through the weighting matrix, W, with added noise, *ϵ* (van der Wilk et al., 2020). The data used to train GP is described as the following:

$$Y=\left[\begin{array}{c} {\hat{f}}_{1},{\hat{\ell }}_{1}\\ {\hat{f}}_{2},{\hat{\ell }}_{2}\\ \vdots \\ {\hat{f}}_{N},{\hat{\ell }}_{N} \end{array}\right]x_{s}=\left[\begin{array}{c} 0\\ c_{1}\\ \vdots \\ c_{N-1} \end{array}\right]\;x_{m}=\left[\begin{array}{c} 0,0,0\\ c_{1},{\hat{f}}_{1},{\hat{\ell }}_{1}\\ \vdots \\ c_{N-1},{\hat{f}}_{N-1},{\hat{\ell }}_{N-1} \end{array}\right]$$

We explored two different input configurations in the study (see Section 3.3.1 for discussion). The first single input configuration, **x**_*s*_, consists of the specified cues at the preceding time step. The second configuration, **x**_*m*_, extends the inputs to include the preceding cadence and stride length. The subscript “s” or “m” indicates single or multi-input configuration. The second configuration aims to improve the model’s prediction performance by conditioning the prediction of the response to cues based on the preceding gait state. The output **Y** consists of ${\hat{f}}_{n}$ , the estimated cadence, and ${\hat{\ell }}_{n}$ , the estimated stride length, at the *n*th step from the gait measurement sub-system. *c*_*n*−1_ is the cue given at the previous step that results in the *n*th cadence/stride length. *n* is incremented at every footstep and *n* = [1, 2, … , *N*]. *n* = 1 represents the baseline cadence and stride length when no cue is given. Sparsity is introduced to the MOGP through inducing points, $Z={\left\{\left[z_{1},z_{2},\ldots z_{M}\right]\right\}}_{d=1}^{D}$ along each input dimension D. Then, the MOGP prior, *p*_0_, can be written in terms of **Z**, where

$$p_{0}\left(g_{q}\right)=N\left(m_{q}\left(Z\right),k_{q}\left(Z,Z^{\prime }\right)\right)$$ (3)

The model is used at run time to predict the resultant stride length and cadence at the *n* + 1 step as a function of the input(s) using Eq 1. As the model is trained online during the experiment, a two-phase behaviour emerges that we call the exploration and the converged phase as explored in previous work (Wu et al., 2021). During the exploration phase, which is set to be the first 2 minutes of the experiment, the GP contains more unexplored regions, resulting in higher model uncertainty. In the converged phase, GP prediction performance and model uncertainty stabilize. The behaviour is further discussed in Section 3.3.3.

#### 2.1.3 Optimize cue provision

We utilize the MOGP model to compute a metronome frequency to minimize the squared difference between the predicted gait state and the desired gait state while suppressing rapid cue changes. The desired gait state consists of a cadence target and a stride length target, which are selected using the process described in Section 2.3. The cost function is defined as the following:

$$\begin{array}{ll} c_{\mathrm{opt}} & =\underset{c_{n}^{⋆}}{arg\;min}\;J\text{, subjected to }c_{\min}\leq c_{n}^{⋆}\leq c_{\max}\\ J\left(c_{n}^{⋆}\right) & =\alpha_{f}{\left(f_{\mathrm{target}}-{\hat{f}}_{n+1}^{⋆}\right)}^{2}+\alpha_{l}{\left(\ell_{\mathrm{target}}-{\hat{\ell }}_{n+1}^{⋆}\right)}^{2}\\ & \;+\alpha_{e}{\left(c_{n}^{⋆}-c_{n-1}\right)}^{2} \end{array}$$ (4)

where *c*_*opt*_ is the optimal metronome frequency subject to the constraints. *f*_*target*_ and *ℓ*_*target*_ are the cadence and stride length targets respectively. *α*_*f*_, *α*_*l*_, and *α*_*e*_ are three scaling factors that weigh the relative importance of each cost term. ${\hat{f}}_{n+1}^{⋆}$ is the predicted cadence and ${\hat{\ell }}_{n+1}^{⋆}$ is the predicted stride length estimated from the MOGP at *n* + 1, given the current cue, $c_{n}^{⋆}$ , or current cadence/SL using Eq. 1. Compared to our previous work in (Wu et al., 2023a) where rapid cue changes are suppressed by changing the constraints based on the current cadence (i.e., *c*_*min*_/*c*_*max*_ = ±20*%f*_*n*_), the difference between the selected cue and the previous cue is added to the cost function. The change aims to address the local minima created due to the algorithm encountering constraints when perhaps providing more extreme cues can lead to greater benefit. The cost term does not prohibit the more extreme cues from being reached like the previous framework but moves gradually toward the desired cue.

### 2.2 Adaptive framework parameters

There are no parameters to tune in the gait estimation algorithm. For the MOGP, the key model parameters are selected to be *Q* = 2, *M* = 20 when *D* = 1 for **x**_*s*_, and *M* = 10 when *D* = 3 for **x**_*m*_. In the study, Q is selected to be 2 as it corresponds to the two output dimensions, which remained constant throughout the experiment. The input dimension D is dictated by the model input structure (i.e., *x*_*s*_ and *x*_*m*_). The number of inducing points, M, is selected based on the time it takes to optimize parameters. *M* is decreased as the dimension of the input increases in **x**_*m*_. The covariance, *k*_*q*_ (⋅, ⋅′), is chosen to be the sum of a squared exponential kernel and a constant kernel. During cue selection, the constraints are *c*_*min*_ = 0.65*f*_*baseline*_ ≤ *c*_*opt*_ ≤ *c*_*max*_ = 1.35*f*_*baseline*_. For the majority of the experiment, we selected *α*_*f*_ = 1.5, *α*_*l*_ = 10, and *α*_*e*_ = 0.05. For the last 4 PD participants, we tested *α*_*l*_ = 20, then *α*_*l*_ = 100 for the last two. The effect of these parameter changes are discussed in Section 3.3.2.

### 2.3 Target selection

**FIGURE 2:**
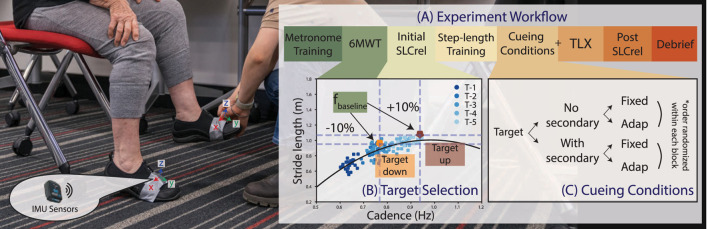
**(A)** The experiment workflow consists of 7 stages as described in Section 2.5. Note: the order of the step-length training and the initial SLCrel test is switched for participants who received the second version of the SLCrel test described in Section 2.5. **(B)** A visualization of the two potential SL/CAD targets derived from the initial SLCrel with respect to *f*_*baseline*_ computed during **6MWT**. **(C)** The main experiment conditions consist of the combination of the two cueing strategies (fixed v. s adaptive) over the two task conditions (no secondary task v. s. with secondary task). The order is block-randomized and blinded from the participants.

Optimizing cue-provision requires setting *f*_*target*_ and *ℓ*_*target*_ in Eq 4, which are set based on the participant’s baseline cadence (*f*_*baseline*_) and initial SLCrel. The full procedure is described in Section 2.5. Here, we describe constructing the **initial SLCrel**, indicated in the experiment workflow diagram (Figure 2A). The initial SLCrel is constructed by playing metronome beats at a fixed pace at 70, 85, 95, 105, and 115 beats per minute (bpm) in random order. 50 beats are provided for each frequency. The range is selected based on Egerton et al. (2011), with the upper range reduced as we intend to provide cues in the region where SLCrel holds. A quadratic and a linear polynomial are fitted to the training data using NumPy (Harris et al., 2020) and the polynomial with a lower residual becomes the SLCrel. An example SLCrel is shown in Figure 2B *ℓ*_*target*_ is selected to be a 0.1 m increase from the SLCrel, which is to ensure that the change is large enough to be detected by the gait estimation algorithm. Two candidate targets (labelled Target up/down in Figure 2B) are evaluated by computing the y-values at ± 10*%f*_*baseline*_ on the SLCrel and adding a 0.1 offset. The up/down target is selected based on the participant’s gait performance during the initial SLCrel measurement. The lower target is chosen if the participant behaves conservatively during training or is unable to follow the faster beats. The lower target is used for 6 out of 13 OA participants and 7 out of 8 PD participants.

### 2.4 Cueing conditions

The main experiment consists of testing two cueing approaches (fixed and adaptive) under two task conditions (no secondary task v. s with a secondary task), as shown in Figure 2C. The cueing approaches are randomized within each task condition and blinded from the participants. The fixed approach provides cues directly at *f*_*target*_, whereas the adaptive cue computes cues based on the framework described in Section 2.1. Both cueing approaches provide cues when the participant’s stride is shorter than the stride length target and provide 10 metronome beats (5 steps per foot). In the adaptive approach, GP is updated after every set of metronome beats and a new cue is computed. For the condition without a secondary task, participants only need to modulate their gait following the auditory cues (see Section 2.5 for further instructions). In the secondary task condition, participants have to recite as many words beginning with a randomly selected letter as possible while walking, which has been used in previous studies (e.g., Lohnes and Earhart, 2011; Beurskens and Bock, 2012). For the experiment, the task is sufficiently challenging, suitable for the experiment duration, and requires no overhead setup.

### 2.5 Protocol

The experiment protocol is shown in Figure 2A. In the text below, key steps that correspond with the workflow are in bold. Participants first watched an introductory video and signed the consent form after having a chance to ask questions. Two IMU sensors were fastened onto the shoes at the crease created by asking the participant to place the foot on the ground and lift the heel, with the orientation shown in Figure 2. Participants went through a **metronome training** session, where a metronome beat was randomly selected and participants were instructed to sync their walking to the beat naturally, one beat per step. Next, participants were told to forget about the pace set during training and we measured their baseline cadence (*f*_*baseline*_) in a 6-min walk test (**6MWT**). The **Initial SLCrel** of the participant is then measured using the procedure described in Section 2.3. Two versions of the SLCrel data collection procedure were administered. From OA#1-11 and PD#1, participants were only asked to sync their walking to the provided beats. Participants then went through **step-length training**, where they were told to first sync naturally to the beat. Keeping to the same beat, they were instructed to take bigger steps and then smaller steps. They were told that the demonstration shows the variety of step lengths one could associate with a beat and their goal during the experiment was to interpret the goal set by the metronome in terms of step length and cadence. They would know if they have it right once the metronome turns off and the goal is to keep the metronome off. The intention of the general instruction is the ability to expand on the framework cost function that may not have a clear physical interpretation (e.g., minimize jerk, which is often seen in tremor suppression (Hu et al., 2019)). In the second version of the SLCrel data collection, the researcher first ran through the step-length training. Then, when the 5 sets of fixed beats were provided, participants were instructed to take 20 natural steps, 20 big steps, and 10 small steps. The process aims to provide a richer training dataset to initialize GP. As these changes in the experimental procedure were made to the ongoing sessions, we did not balance the number of participants in each group. This is a limitation of the study, and is discussed further in Section 4.

After the demonstration/SLCrel data collection, participants went through a practice session, where the experimenter played the role of the metronome system with the goal of decreasing the participant’s stride length (i.e., opposite of the main experiment). The participant was first told to walk naturally at the start of the practice. After a few steps, the experimenter manually played a metronome beat and the participant was guided to change their step lengths. Once the experimenter saw the participant taking smaller steps consistently, the beat was turned off. The experimenter then reminded the participant to try and develop a strategy to turn the metronome off and keep it off. Participants then went through the first two **cueing conditions** where there was no secondary task. Before commencing the conditions with the secondary task, participants were reminded that they still had to keep the metronome off while reciting words and the two tasks were equally important. After each experiment condition, participants answered a custom survey and a NASA-Task Load Index (**TLX**). After completing all 4 experiment conditions, participants repeated the SLCrel data collection (**Post SLCrel**). A final survey was administered to collect information about the participants’ overall experience. Participants were then given a chance to review their data and be informed of the cueing conditions they performed during the experiment. The project was approved by Monash University Human Research Ethics Committee (ID 37639) and Monash Health Human Research Ethics Committee (RES-22-0000-516A).

### 2.6 Materials

A Python program was developed for data collection and runs on a Windows 10 Laptop (i5 core with no GPU). The program interfaces with the wireless IMU sensors (WaveTrack Inertial System, Cometa Systems, Milan, IT) and controls the timing of the auditory cues. The cues are played from a Philips speaker (BT50A), which is connected to the computer via a 3.5 mm audio cable. The GP model was implemented using GPFlow 2.8 (van der Wilk et al., 2020) and the cost function was solved using the Nelder Mead Method in Scipy 1.7.1 (Virtanen et al., 2020).

### 2.7 Participants

A total of 25 participants enrolled in the study. The two recruitment channels of the study consisted of the City of Monash volunteer group and the clinicians at Monash Health. All the OA in the study were recruited from the volunteer group; 7 PwP enrolled through clinician referral and one PwP joined from the volunteer group. OA were eligible if they were over 60 years old and had no issues with walking, balancing, or hearing. PwP needed to have Hoehn and Yahr score ≤2 regardless of medication. The clinical scale is used to classify the motor function of Parkinson’s Disease (Bhidayasiri and Tarsy, 2012). Participants with a score of ≤2 can perform their daily routine independently or with minimal assistance. For potential PwP enrolling without clinician referral, we developed a list of questions based on the Hoehn and Yahr scale and asked the participant to self-assess their motor performance. The participant had no issue with walking, balancing, or performing their daily tasks and therefore was enrolled in the study. All participants were tested during their medication-ON state (if they were on medication). A few PD participants took medicine during the experiment. We did not collect data on the type/frequency of their medication. While 25 participants took part in the study, data from 21 participants were used in the analysis. This is further discussed in Section 3.1.

### 2.8 Analysis

Two separate analyses were conducted with respect to the two aims of the study.

#### 2.8.1 Evaluate overall performance: cueing approach, participant group, task condition

First, we considered the following research questions.

• RQ1: Did the fixed and adaptive cueing strategies differ in their effectiveness in modifying participants’ gait?
• RQ2: Did the participant group impact responsiveness to cues?
• RQ3: How did secondary task affect cue responsiveness?

The research questions are examined in detail in Section 3.2 by computing the change in stride length from baseline, change in cadence from baseline, cadence target error, and the percentage of time cue is required. The TLX score is also reported to capture the participants’ perceptions. Finally, the pre and post-stride lengths from the SLCrel tests were compared.

The gait performance metrics (i.e., change in SL and cadence) were analyzed using t-tests and Linear Mixed Effect (LME) model. *t*-test of each individual’s gait data for every cueing condition is compared to the individual’s baseline data to determine the effect size of the cueing condition. The test would show the effect of the cueing condition on the individual level. As there is limited space to display the sample mean for all 84 experiment conditions, we reported the significance in terms of the number of significant/non-significant instances and the population mean. The number of samples included for each cueing condition focused on the converged phase (i.e., the number of steps taken in the last 2 min of the experiment). The number of samples from the baseline condition matched the number of steps in the converged phase (roughly matching the last 2 min during baseline to the last 2 min during each experiment). The total number of samples was different for each participant, ranging from 80 to 100+ samples per group.

After comparing the difference to baseline, LME models were used to investigate the effect of the three independent variables (i.e., cueing approach, participant group, and task condition), which were included as the fixed effects. The LME model is different from the *t*-test results as it shows the average effect over the sample population. Specifically, the cueing approach effect consists of fixed and adaptive approaches; the participant group is the divide between OA and PD^1^; and the task condition is walking with or without secondary task. LME models can account for the between- and within-subject variability introduced through repeated measures. Normality and homoscedasticity assumptions were checked using Shapiro-Wilk test and Levene test respectively. Since the order of the experiment conditions was randomized, it was not included as a fixed effect. The LME model that had all the fixed effects and the three-way interaction was first built using lme4 and lmerTest in R (Bates et al., 2015; Kuznetsova et al., 2017). The model was subsequently modified by removing the combination of interaction terms with no significance until the model only contained fixed effects. Only significant results are shown in the figures. Additional statistics are reported using marginal means (*μ*_*em*_), which is the mean of the group when averaging over the fixed effect(s), or differences between marginal means (Δ*μ*_*em*_). We also reported the intraclass correlation coefficient (ICC), which is the percentage of the variance due to individuals over the total variance of the random effect. The analysis of the gait metrics focused on the converged phase (i.e., the last 2 minutes of each experiment). Since the exploration phase is considered a transient phase, the analysis examines the steady-state behaviour that is the converged phase.

#### 2.8.2 Evaluate adaptive framework performance: input structure, training procedure, convergence, and model structure

We conducted four separate analyses to study the behaviour of the GP model. In the first analysis, we evaluated the GP model performance with respect to the parameters, including the input structure, training procedure, and model structure. For evaluating the effect of model input (*x*_*s*_ *versus* *x*_*m*_) and the effect of the training procedure (Procedure 1 *versus* 2), we performed an analysis by computing the mean squared error (MSE) and the standard error (SE) for every participant for the adaptive experiment condition with no secondary task and trained the GP models again with all the data. In this exploratory study, the aforementioned analysis was conducted after the first 12 participants (11 OA and 1 PwP), which prompted the switch to *x*_*m*_ and Procedure 2 for the remaining 8 participants (1 OA and 7 PwP). These changes were better made during the study than *post hoc* because the outcome metrics depend on the participants’ reactions and cannot be re-created after the experiment.

In the second part of the analysis, we visualized all the PwP performance metrics grouped by the parameter choices was examined to determine whether the changes to the experiment procedure/GP modeling choices correlate with performance gain.

In the third part of the analysis, we evaluated the convergence of the GP model using the Kullback-Leibler (KL) divergence. KL divergence helped quantify the difference between the GP model at each training iteration and the final model using the closed-form solution from Robert (1996) summed over the input range. The *x*_*m*_ GP input structure was used for the analysis. The KL analysis provided evidence of GP convergence to support using the halfway point in the experiment (i.e. 2 min) to separate the exploration and converged phases.

Finally, to evaluate the GP model structure, we computed the Akaike information criterion (AIC) and Bayesian information criterion (BIC) over three model variations. While both AIC and BIC represent a tradeoff between model complexity and goodness of fit, BIC penalizes the model complexity more heavily as it is scaled by the number of data points. A model with the lowest AIC and BIC would represent the best tradeoff between model accuracy and complexity.

## 3 Results

The result section is split into three parts. Section 3.1 presents the demographic of the participants. Section 3.2 answers the research questions with respect to the independent variables. Finally, Section 3.3 examines the impact of the changes in the pre-experiment training procedure and the performance of the GP model.

### 3.1 Demographics

17 OA participated in the study (9M/8F, Mean ± Standard Deviation: Age 72 ± 9 years old, Height 167.06 ± 10.82 [cm], Weight 71.86 ± 12.77 [kg]). Four OA were excluded from the analysis: 1 participant was used to refine the study protocol, 1 participant did not finish the study, the gait detection algorithm did not work on 1 participant, and 1 did not meet the age requirement. The gait detection algorithm did not function properly as the participant had a “stiff” walking pattern (walking without bending the knee) due to other lower leg injuries. This caused a breakdown of the stance/swing phases detection and the gait detection algorithm consistently underestimating the footsteps.

Eight PwP joined the study (6M/2F, Age 70 ± 7, Height 173.98 ± 11.60, 73 ± 15.85). The average time since the diagnosis is 6 ± 3.5 years. PwP reported the following lower leg symptoms (the number of participants who reported in brackets): stiffness 1), slowed movement 4), trouble balancing 5), freezing of gait 2), and dyskinesia 2). One participant did not have any symptoms. No PwP had prior experience with cueing using wearable devices. One participant was going through gait rehabilitation. Most participants performed their daily tasks independently, with a few needing help sometimes when dressing, walking, climbing stairs, and doing housework. Overall, data from 21 participants (13 OA and 8 PwP) were included in the following analysis.

### 3.2 Results of overall performance

#### 3.2.1 Change in stride length (SL) from baseline

We computed the participants’ change in SL during the experiments (i.e., the 4 cueing conditions in Figure 2C) compared to their baseline SL measured in the 6MWT as the primary outcome. The metric is in centimeters (cm) and should be as large as possible as the cues aim to lengthen participants’ strides.

The SL during the converged phase was compared to baseline SL for each participant per condition using a *t* -test. The results are summarized in Table 1. The results showed that the SL changed significantly from the baseline for 81% of the participants using the fixed condition for both task conditions. The adaptive approach also changed the baseline significantly for 67% of the participants in the condition without secondary task and 61% of the participants in the condition with secondary task. These results indicate differences from baseline on the individual level. The mean SL across all participants at baseline is 115 cm, and the mean SL across all participants and all cueing conditions is 121cm, which is an average of 6 cm difference.

**TABLE 1:** Number of Participants with Significant/Non-significant Change in SL compared to Baseline.

	Significant (percentage)	Not significant (#OA/#PwP)
Fixed-no task	17 (81%)	4 (2 OA/2 PwP))
Adap-no task	14 (67%)	7 (5/2)
Fixed-with task	17 (81%)	4 (2/2)
Adap-with task	13 (62%)	8 (5/3)

Fixed/Adap indicates the cueing conditions. No task/with task indicates the condition without/with secondary task. The total number of participants across each row is 21 (13 OA/8PwP).

**FIGURE 3:**
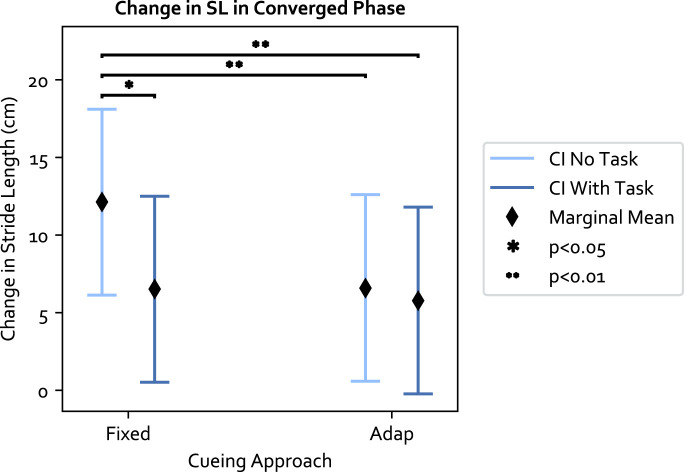
The marginal means of the cueing approaches and 95% confidence interval (CI) in relation to the interaction. Within each cueing approach, the decrease in SL in the fixed approach between the no secondary task (no task) v. s with secondary task (task) condition is significant. The adaptive approach has similar performance across the task condition.

Next, an LME model was formulated considering the influence of the cueing approach, participant group, and task condition. The LME model residual of the 84 trials was first checked for the normality and homoscedasticity assumptions. The model passed both the Shiparo-Wilk test (*p* = 0.29 > 0.05) and Levene test (*p* = 0.77 > 0.05). The LME model showed a significant interaction effect between the cueing approach and the task condition (F (1,60) = 4.090, *p* = 0.048, Value = 4.799, Standard Error (SE) = 2.373, 95% Confidence Interval (CI) = [0.190, 9.409]). This meant that the stride length change induced by the fixed approach was significantly different between the condition with v. s without secondary task. Specifically, the marginal mean of the fixed approach with secondary task dropped by 5.61 cm compared to no secondary task (Δ*μ*_*em*_ = 5.61 *cm*). On the other hand, the performance of the adaptive approach was not significantly different between task conditions (Δ*μ*_*em*_ = 0.82 *cm*). The ICC was 82%, indicating large individual differences. The resulting marginal mean is shown in Figure 3.

With respect to the three independent variables (cueing approach, participant group, and task condition): 1) the fixed approach was more effective than the adaptive approach without secondary task. The adaptive approach maintained the same level of performance regardless of the task condition; 2) the secondary task shortened SL to a similar extent in both OA and PwP (Δ*μ*_*em*_ = 3.22 *cm*), and 3) PwP increased their SL more than OA (Δ*μ*_*em*_ = 9.75 *cm*), though the effect was not significant.

#### 3.2.2 Change in cadence from baseline and cadence target root-mean-square-error (RMSE)

We first examined whether the cadence significantly changed from the baseline. The result is shown in Table 2. Overall, the mean cadence across the population is 0.873 Hz. The mean cadence across all cueing conditions is 0.831 Hz, which is an overall 0.042 Hz decrease from baseline. The fixed condition without secondary task was able to induce a change in cadence for all the participants; the effectiveness dropped slightly in the presence of secondary task. The adaptive approach induced a similar level of change in cadence across the two task conditions.

**TABLE 2:** Number of Participants with Significant/Non-significant Change in Cadence compared to Baseline.

	Significant (percentage)	Not significant (#OA/#PwP)
Fixed-no task	21 (100%)	0 (0 OA/0 PwP))
Adap-no task	18 (86%)	3 (2/1)
Fixed-with task	20 (95%)	1 (1/0)
Adap-with task	18 (86%)	3 (1/2)

Fixed/Adap indicates the cueing conditions. No task/with task indicates the condition without/with secondary task. The total number of participants across each row is 21 (13 OA/8PwP).

**FIGURE 4:**
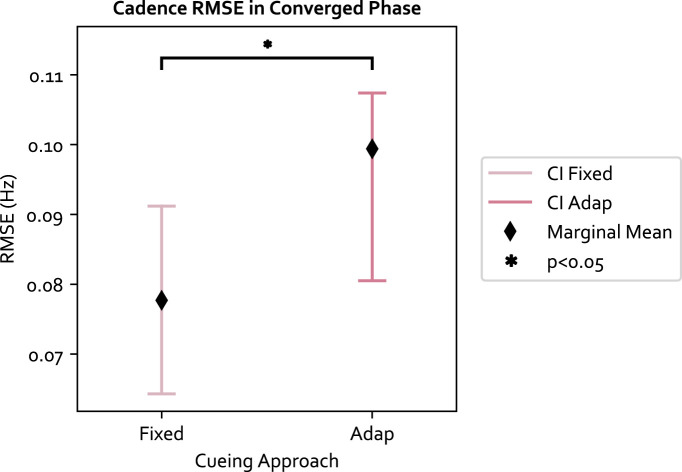
The cadence RMSE for each cueing approach and 95% confidence interval (CI). The fixed approach has a lower RMSE compared to adaptive, which is expected since the fixed approach provides cues directly at the target.

**FIGURE 5:**
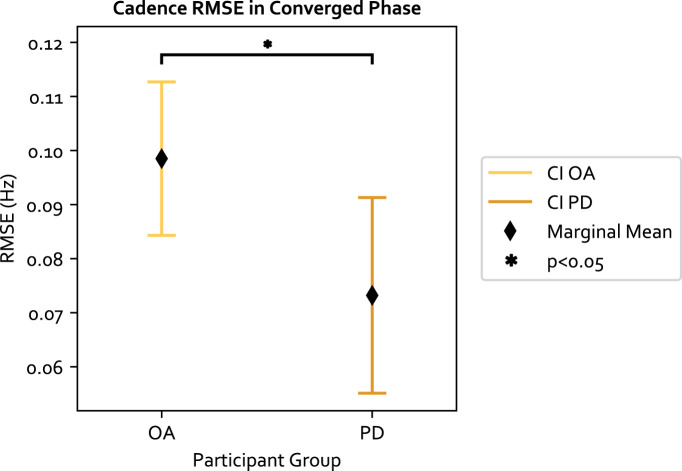
The cadence RMSE across the two groups of participants and 95% confidence interval (CI). PD participants have a lower RMSE compared to OA.

Moreover, we examined the cadence target RMSE, which measures how well the target was achieved. The cadence target RMSE is a secondary performance measurement that examines how well the target selected from the original SLCrel can be maintained when stride length was changing. A low RMSE indicates participants were able to modulate their SL without changing the cadence. The LME model residual of the 84 data points passed the Shiparo test (*p* = 0.85) and Levene test (*p* = 0.32). With respect to the three independent variables: 1) the adaptive approach was significantly worse than fixed (F (1, 61) = 4.791, *p* = 0.032, Value = 0.016, SE = 0.0074, CI = [0.002, 0.031]), illustrated in Figure 4. The “Value” number indicates the LME model predicts a 0.016 Hz increase in RMSE when using the adaptive approach compared to the fixed. The result was expected as the fixed approach provides cues directly at the target cadence, which was not the case for the adaptive approach; 2) The secondary task increased the target RMSE but was not significant, and 3) PwP had a significantly lower cadence RMSE compared to OA (F (1, 19) = 5.310, Value = −0.025, SE = 0.011, CI = [-0.047, −0.004]), as shown in Figure 5. The ICC was 21%, which means there is a lower individual difference compared to the change in SL.

#### 3.2.3 Percent on

The percent on metric computes the percentage of time the cue was played during the experiment. A lower number indicates better cueing efficiency, as fewer cues are needed to increase the stride. The model residual of the 84 data points passed the normality test (*p* = 0.30) and Levene test (*p* = 0.37). We observed with respect to the three independent variables: 1) the adaptive approach played cues 2.85% more than the fixed approach 2) the secondary task also caused a 2.65% increase in cue-playing and 3) PwP required 7% less cues than OA. These differences are not statistically significant.

#### 3.2.4 Task Load Index (TLX)

**FIGURE 6:**
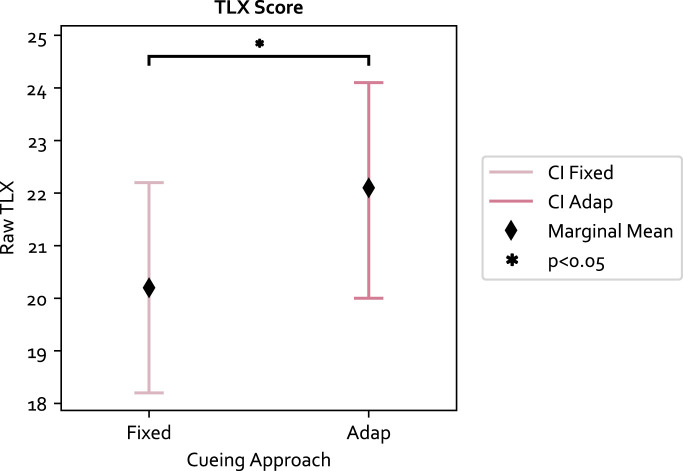
The TLX score is significantly higher in the adaptive approach.

**FIGURE 7:**
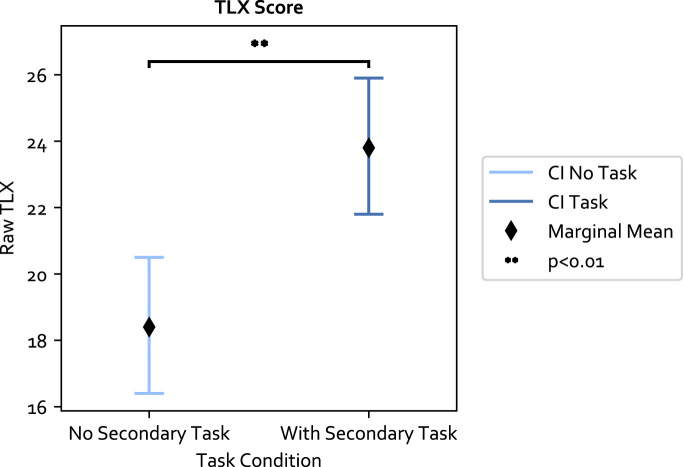
The secondary task increases the mental workload significantly.

The raw TLX score was computed for each experiment condition. The LME model residual of the 84 data points passed the normality test (*p* = 0.79) and Levene test (*p* = 0.33). The results with respect to the three independent variables are as follows: 1) the secondary task significantly increased the mental workload and had the biggest effect size among the three variables (F (1, 61) = 38.78, *p* ≪0.05, Value = 5.404, SE = 0.868, CI = [3.705, 7.105]); 2) the adaptive approach also significantly increased the mental workload compared to the fixed approach (F (1, 61) = 4.70, *p* = 0.034, Value = 1.88, SE = 0.868, CI = [0.181, 3.581]); and 3) the TLX score was not significantly different between PwP and OA. The results are shown in Figures 6, 7. The ICC was 41%, indicating large individual variation.

#### 3.2.5 Pre/post stride length change

To evaluate whether the cueing conditions had prolonged effects on participants’ gait, the pre-post SL performance was compared using a paired *t*-test at each of the 5 beats provided. The normality assumption was validated using the Shapiro-Wilk test for each of the 5 beats and all beats passed the test (*p* = 0.57, 0.07, 0.99, 0.46, 0.47 from the slowest to the fastest beat respectively). The pre/post group each contains 21 data points. No statistical significance in SL change was found at the slowest and fastest fixed metronome pace (70&115 bpm). An increase was seen between the pre and post-SL measurements for all intermediate beats (85, 95, 105 bpm). At 85 bpm, the mean SL increased by 6.61 cm (mean difference M) = 6.61, standard deviation (SD) = 10.64 cm, t-value *t*) = 2.847, *p* = 0.010). At 95 bpm, M = 6.05, SD = 9.77, *t* = 2.837, *p* = 0.010. Finally, M = 4.77, SD = 6.97, *t* = 3.138, *p* = 0.005 for 105 bpm. In the post-test measurement, participants repeated the initial SLCrel data collection procedure (i.e., sync naturally or natural syncing plus taking big/small steps). The data from participants who were instructed to take big/small steps were excluded (i.e., only including the natural steps). An increase in SL in the intermediate beats demonstrated that there was a carryover effect from the experiment, as participants continued to lengthen their strides despite being told to sync to the beats naturally.

### 3.3 Results of adaptive framework performance

The following section focuses on studying the performance of the GP model in relation to the experiment procedure and modeling choices.

#### 3.3.1 Input data and training procedure

##### 3.3.1.1 Input data

We first compared the performance between the input formulations (**x**_*s*_ and **x**_*m*_) discussed in Section 2.1.2. The MSE is the average prediction error and the SE indicates whether the prediction error is within the GP’s variance (SE < 1 indicates the error is within the GP variance). During the experiment, the analysis was conducted after the first 12 participants (11 OA/1PwP), which prompted the change in model structure for the remaining participants. In this *post hoc* analysis, since information regarding the previous gait state was recorded, data from all participants (number of participants, n = 21) were used to compute the MSE/SE for *x*_*s*_ and *x*_*m*_. The MSE for **x**_*s*_ was 0.007 Hz^2^ and 0.036 m^2^ for cadence and SL respectively. The MSE was lowered to 0.005 Hz^2^ and 0.021 m^2^ using **x**_*m*_. This meant adding more information about the previous gait state improves GP prediction. The improvement was small for cadence as the cues could entrain cadence. The benefit of extending the input states was seen in the SL prediction, where the average error was reduced by 0.015 m^2^.

##### 3.3.1.2 Training data

The next analysis examined whether more training data improves GP performance. The MSE and SE were computed to evaluate the loss on the dataset under three scenarios: using the initial SLCrel data only (referred to as training only), using data collected during the experiment only (i.e., testing only), and using both (training + testing). Data from the no-task condition was used. We proceeded with using **x**_*m*_ as the formulation. During the experiment, all participants used both the training and testing data. In this analysis, all participants were also used to compute the MSE/SE (n = 21). The results are presented in Table 3, The MSE and SE were the highest for both CAD and SL when GP was presented with only the training data as shown in Table 3. This demonstrated the importance of online learning as the new data can help the model adapt to the varying gait performance. Between using testing data (second row) *versus* training + testing (third row), both SL and cadence (CAD) prediction MSE were similar. However, the SE was smaller when using both training and testing data. The use of the testing data provided more diverse samples to the GP model, thereby reducing the variance in regions where GP might not have explored during the regimented training phase.

**TABLE 3:** Comparison between input data on GP Performance.

Input data	Metric	MSE	SE
Training only n = 21	CAD	0.0269	0.5045
	SL	0.0973	0.8903
Testing only n = 21	CAD	0.0053	0.4786
	SL	0.0196	0.8733
Training + Testing n = 21	CAD	0.0054	0.3618
	SL	0.0205	0.6750

The unit of cadence mean squared error (MSE) is *Hz* ^2^ and the stride length MSE, is *m* ^2^. The unit on cadence standard error (SE) is *Hz* ^2^ and SL SE, is *m*. The number of participant n) is indicated for each row.

##### 3.3.1.3 Training procedure

Finally, we examined the GP performance between the two initialization procedures (i.e., the two different SLCrel collection procedures described in Section 2.5). In the first procedure, participants were told to sync to the beats normally. In the second procedure, participants were told to take neutral, bigger, and smaller steps while syncing to the fixed beats. We compared the difference when using the **x**_*m*_ and using both training + testing data. The result is presented in Table 4. The prediction MSE did not change much between the two procedures, meaning the training procedure did not affect the GP’s mean. However, the second procedure, which was designed to mimic the experiment condition, improved the GP performance as it reduced the standard error, particularly for the SL prediction. During the experiment, it was hypothesized that changing the experimental procedure can improve the adaptive framework prediction, prompting the change in the protocol after the initial 12 participants (11 OA/1PwP). As *post hoc* analysis cannot make up for the missing big/small steps recorded in Procedure 2, the analysis divided the result based on the procedure.

**TABLE 4:** Comparison between training procedure on GP Performance.

Input data	Metric	MSE	SE
First procedure n = 12 (11 OA/1 PwP)	CAD	0.005	0.381
	SL	0.022	0.740
Second procedure n = 9 (2 OA/1 PwP)	CAD	0.006	0.345
	SL	0.019	0.590

The unit of cadence mean squared error (MSE) is *Hz* ^2^ and the stride length MSE, is *m* ^2^. The unit on cadence standard error (SE) is *Hz* ^2^ and SL SE, is *m*. The total number of participant for each group (n), along with the breakdown of participant is shown in each row.

#### 3.3.2 Impact of *post hoc* choices on performance metric

**FIGURE 8:**
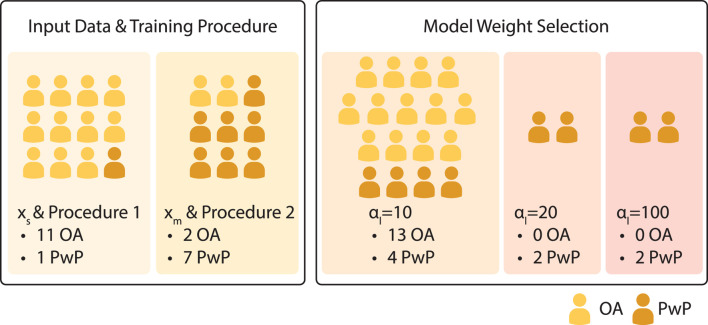
Participant grouping with respect to changes to the model parameters/experiment procedure during the experiment.

**FIGURE 9:**
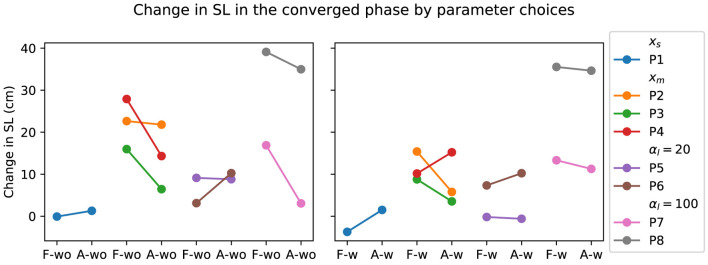
Visualization of PwP SL change compared to baseline grouped by the parameter choices. *x*_*s*_ indicates the group where the GP model structure where a single input is used to predict the two outputs. *x*_*m*_ is the GP formulation where three inputs are used to predict the two outputs. *α*_*l*_ = 20 is the group where the SL cost term is 20. *α*_*l*_ = 100 is the group where the SL cost term is further increased to 100.

**FIGURE 10:**
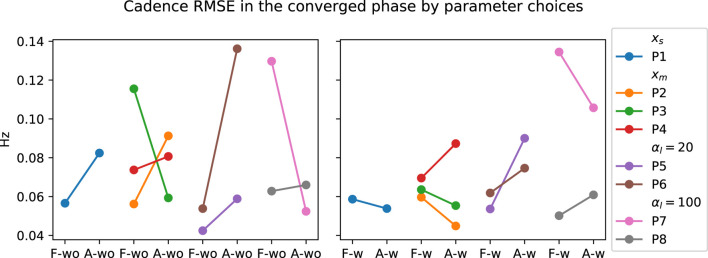
Visualization of PwP’s cadence RMSE grouped by the parameter choices. *x*_*s*_ indicates the group where the GP model structure where a single input is used to predict the two outputs. *x*_*m*_ is the GP formulation where three inputs are used to predict the two outputs. *α*_*l*_ =20 is the group where the SL cost term is 20. *α*_*l*_ = 100 is the group where the SL cost term is further increased to 100.

**FIGURE 11:**
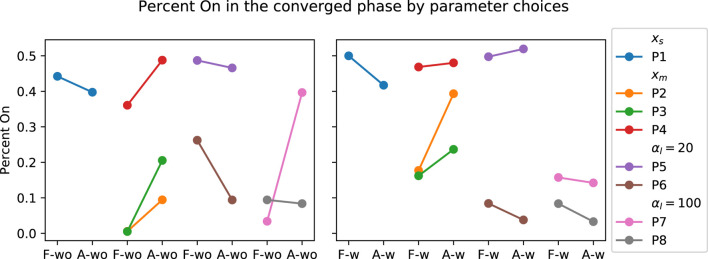
Visualization of PwP’s percent on time grouped by the parameter choices. *x*_*s*_ indicates the group where the GP model structure where a single input is used to predict the two outputs. *x*_*m*_ is the GP formulation where three inputs are used to predict the two outputs. *α*_*l*_ = 20 is the group where the SL cost term is 20. *α*_*l*_ = 100 is the group where the SL cost term is further increased to 100.

**FIGURE 12:**
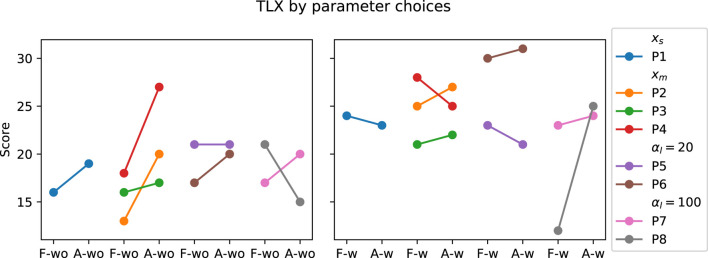
TLX Score of PwP grouped by the parameter choices. *x*_*s*_ indicates the group where the GP model structure where a single input is used to predict the two outputs. *x*_*m*_ is the GP formulation where three inputs are used to predict the two outputs. *α*_*l*_ = 20 is the group where the SL cost term is 20. *α*_*l*_ = 100 is the group where the SL cost term is further increased to 100.

In this section, the effect of the parameter changes during the study was visualized and examined. The analysis helped determine whether the parameter changes introduced factors that may have impacted the outcome. The changes include the choice of the cost function weight (*α*_*l*_), as discussed in Section 2.2, and the model structure selection (*x*_*s*_ *versus* *x*_*m*_) and training procedure (Procedure 1 *versus* 2), discussed in Section 3.3.1. The participant grouping for the corresponding parameter changes during the experiment is shown in Figure 8. The change in SL grouped by parameter choices for the PwP data is presented in Figure 9; cadence RMSE is in Figure 10; percent on time is in Figure 11, and finally the TLX score is in Figure 12.

Data from PwP were plotted for visualization as the group experienced all the parameter changes. From the visualization, no apparent link was identified between the changes in framework parameters to the performance metric. Instead, other factors that were identified to have a larger impact on the performance metrics, including data availability (i.e., having sufficient data and variability to cover the larger state space as discussed in Section 3.3.1) and the stability of the cues (i.e., participants found the target difficult to figure out as discussed later in Section 4).

#### 3.3.3 Exploration and converged phases

**FIGURE 13:**
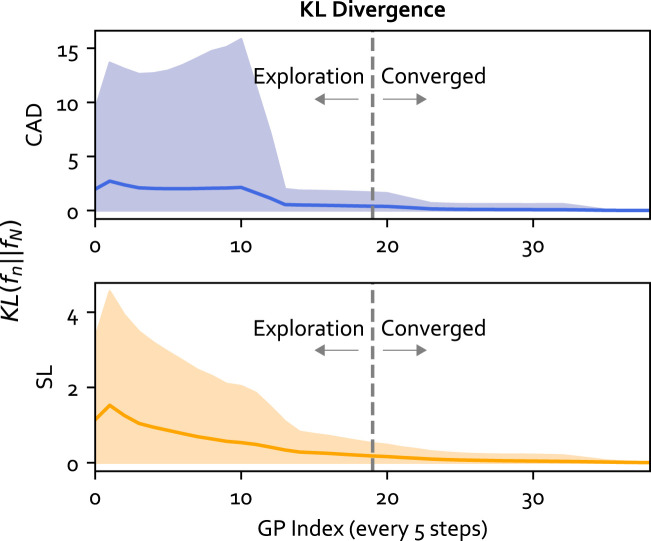
The KL divergence for the GP model at each training iteration (every 5 steps) compared to the final GP model. The mean for each output is plotted as a solid line and the standard deviation is plotted as the shaded area. The vertical line separates the exploration and converged phases.

In this section, the assumption of using the halfway point in the experiment (i.e. 2 min) to separate the exploration and converged phases of GP is examined using KL divergence. KL divergence represents the relative entropy between two distributions, meaning the metric can be used to quantify the amount of information gained from continuous learning. The result averaged over all participants is shown in Figure 13. From the figure, the KL for cadence (purple) and stride length (yellow) were high initially. This is because various cues were provided during the experiment compared to training where cues at fixed rates were provided, allowing GP to better model the person’s response to cues through online learning. During exploration, it was observed that the KL decreased faster, while in the converged phase the learning rate plateaus. This means the GP model converges to a steady state performance during the experiment.

#### 3.3.4 Model structure selection

**FIGURE 14:**
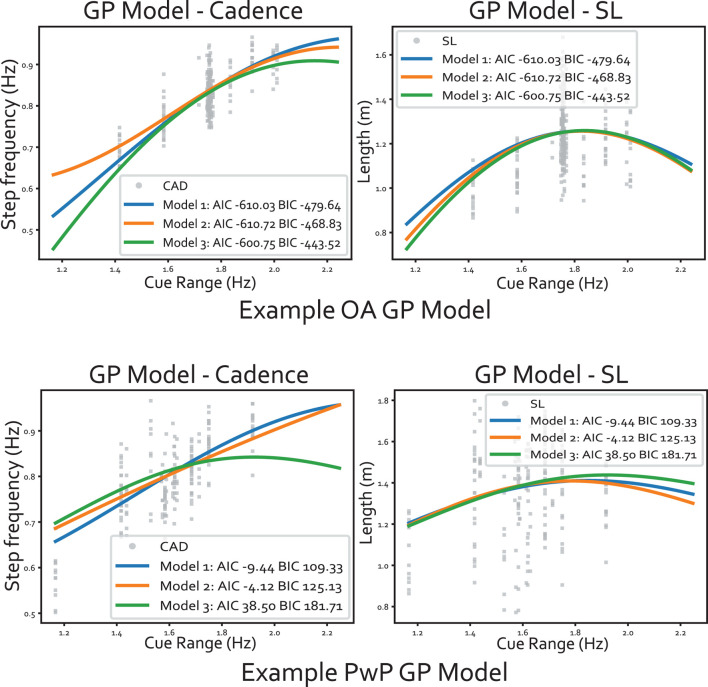
Example of the GP model fitted to the data, where the input dimension is flattened into 1D (along the range of cues). The panels at the top show an example of data from an older adult and the bottom panels are from a PD participant. Within each panel, the output cadence is plotted on the left and the stride length is on the right.

In the experiment, we adopted the GP model with two independent kernels and a correlation matrix as outlined in Section 2.1.2. The section examines the choice of GP structure by comparing the AIC and BIC of 3 model structures. **Model 1** represents the most rudimentary version of the Sparse MOGP, with a kernel shared between the two outputs (i.e., q = Q = 1) and no correlation matrix W. **Model 2** comprises independent kernels (Q = 2) with no correlation matrix. **Model 3** is the full GP structure described in Section 2.1.2. The result is summarized in Table 5 and examples of the model fit are shown in Figure 14. The goal is to minimize both the AIC and BIC scores. When looking at the visualization, all GP models had a similar mean in the data-rich region, which explained the similarity in the AIC/BIC scores in Table 5. A bigger difference was seen towards the tail-ends of the model where there was a lack of data.

**TABLE 5:** Metrics for model structure selection.

	AIC	BIC
Model 1	−463.721	−340.101
Model 2	−488.233	−353.705
Model 3	−434.851	−285.779

Based on the metrics, Model 2 had the lowest AIC and BIC, meaning the correlation matrix was not essential but having the independent kernel helped. The two independent kernels enabled the GP to capture differences in the variation magnitude between the two outputs. Figure 14 illustrates the GP models where the input dimension is collapsed into 1D over the range of cues. We can see the data spread for cadence is smaller than the spread for SL. The difference in data spread is especially prominent for the PwP GP model. For instance, at around 1.4 Hz, the cadence ranges from 0.65-0.85, but SL varies from 0.8-1.8. Therefore, the different kernels along each output dimension would better model this behaviour. The larger spread in SL aligned with the training procedure during the experiment, as the instruction specifically requested participants to try various stride lengths at the same cadence. Finally, the correlation matrix W was not shown to be necessary based on the AIC/BIC scores.

## 4 Discussion

In this study, we showed that RAS can be used to change both the cadence and the SL of the majority of the OA and PwP in the current experiment under single and dual-task conditions. The discussion is structured with respect to the three independent variables, starting with the participant group. We also provided a summary of the GP model findings.

**Older Adults compared to PwP**: For the PwP considered in this study, there was no statistical difference between OA and PwP in terms of their ability to modify their stride length. Previous systematic reviews that examine the effect of RAS in OA and PwP both suggest a small effect size on stride length (Ghai et al., 2018a; Ghai et al., 2018b). Hence, the lack of statistical significance in the stride length change is consistent with the literature. The only significant difference between OA and PwP was seen in the target cadence RMSE, where PwP had a significantly lower RMSE than OA, suggesting that PwP followed the beats more rigorously than OA. This difference may be explained by motivation, where participants with gait impairments (compared to healthy OA) were more willing to change and improve their gait. The result is consistent with a previous study, where PD participants synchronized to the beats more compared to OA (Dotov et al., 2017).

**Fixed compared to Adaptive approach**: In this study, we found that auditory cues can be used to increase the majority of participants’ stride length compared to the baseline. The fixed approach worked for a larger percentage of the sample population compared to the adaptive approach in both task conditions. When examining the cueing approaches on the population level using the LME model, the range of SL changes fell between 5–12 cm (Figure 3). These values are similar to the range reported in previous literature that used fixed cues (e.g., Suteerawattananon et al., 2004; Nieuwboer et al., 2007). This means the adaptive approach, while in general inducing a smaller change in SL than the fixed approach with no secondary task, was still comparable to the ranges reported in previous literature. Reasons for the adaptive approach’s lesser performance may be due to the non-static cues combined with the experiment goal. As participants needed to adjust for cadence (as per the instruction to sync the walking to the cues) and stride length (instructed to explore different step lengths with each pace), it was common for participants to first match their walking pace to the beats, then work on changing their strides after realizing only matching the cadence was insufficient. As one participant put it “[I am] aware of the pace first instead of the step length.“. This means while the cadence adaptation was fast, the change in stride length took longer and required more effort. The difference in difficulty in adjusting cadence *versus* stride length may also explain the percentage of participants with significant change from baseline for cadence was higher than the stride length percentage (i.e., Table 1; Table 2).

**Influence of Secondary Task**: The last independent variable is the presence of the secondary task. In a previous systematic review, dual-task (i.e., RAS + secondary task) conditions have a small effect size on increasing stride length and cadence compared to baseline in PwP (Ghai et al., 2018b). We also observed an increase in SL compared to baseline in both OA and PwP with the word-reciting task on the individual level, though the number of significant changes dropped with secondary task. The cadence RMSE between with and without secondary task is not significant over the sample population. In the post-study interview, participants uniformly agreed that the secondary task was difficult and required more mental effort (as indicated by the TLX score). Four PwP explicitly mentioned they cannot perform beat-following and word-reciting simultaneously and they need to alternate between the two tasks (i.e., task-switch). The benefit of the adaptive approach was more prominent in the task-switching context as the change in pace was noticed more often. The effect was also mentioned in the post-study interviews. While participants frequently associated the adaptive approach with being more “frustrating” than the fixed approach, they also described the adaptive approach as being more “attention-grabbing”, especially in the dual-task condition. Two common sources of frustration when using the adaptive approach came from having difficulties in figuring out the gait targets as participants often thought each new metronome pace meant a different set of targets. In reality, the cues were changing due to the change in the GP model as described previously. The second source of frustration was the adaptive approach being seemingly more persistent. As one participant described it, “the [adaptive approach] really wants me to take bigger steps”. Participants also attributed more personality to the adaptive approach such as saying “the metronome knows when [they’re] slacking” or “the metronome is more assertive”, contrasting the fixed approach which is described as “easy” or “do not have to think much about it”. The perception raises an interesting challenge for RAS design as the fixed RAS may be easily ignored, but too much variation can also cause confusion and frustration. For the current adaptive system, issues with cue variation may be addressed by having a better training dataset and updating GP less frequently.

**Lessons on Adaptive Framework Design**: In this study, the parameters of the adaptive framework were changed during the experiment to better characterize the performance of the adaptive approach. Some changes were more beneficial than others. For instance, as seen in Section 3.3.2, changes to the cost function weights did not improve the participants’ SL change. Instead, more fundamental modeling problems needed to be resolved first, including improving GP prediction accuracy and uncertainty modeling. Factors that improved GP performance include adding information regarding the previous gait states, and having more variety in the training data. Interestingly, the correlation matrix W that related cadence and SL was not found to be beneficial. The correlation matrix may not be necessary as most variation could be captured independently using the GP without mixing the two outputs. The lack of correlation between stride length and cadence was consistent with the literature, where the SLCrel typically observed when participants walk at self-selected paces disappears when either cadence or stride length was constrained (Egerton et al., 2011).

**Limitations of the Study**: There were several limitations of the study. First, for the analysis of how the training procedure and GP model parameters influence the adaptive method performance, the number of participants was not balanced across each group. This presents a potential confound for the adaptive condition results. While we did not find statistically significant differences between participant groups within the adaptive condition, this finding may not generalize and needs to be verified with a follow-up study with balanced participants groups. Second, the gait monitoring algorithm was unable to handle the participant with an abnormal gait pattern and an alternative algorithm should be explored. Third, since the GP performance was influenced by the diversity of the initial training data, more robust techniques could be used to better sample the state space. Previous literature has approached the exploration/exploitation problem using expected improvement (e.g., Kim et al., 2017) and might provide a good starting point. As mentioned previously, less frequent GP updates may also reduce cue variation. Finally, the single-session study setup was insufficient for evaluating habituation or the effect of the medication cycle.

## 5 Conclusion and future work

In this study, we evaluated the performance of an adaptive cue-provision framework that can modulate stride length and cadence using auditory cues. The adaptive framework monitors gait performance using a single IMU sensor and builds an individualized cue-response model to relate how the gait performance changes as a function of the input cues. The cue-response model is then used in an optimization algorithm to determine the cue to provide. The adaptive framework was compared against the state-of-the-art, fixed cue approach where static cues are provided. The two cueing approaches were compared with OA and PwP under two task conditions, with and without secondary task. The result showed that over half of the OA and PwP can modify their stride length and cadence using auditory cues. When no secondary task was present, the fixed approach was the best at increasing stride length, but the performance of the fixed approach dropped significantly in the presence of the secondary task. The benefit of the adaptive approach was its ability to maintain the same level of stride length change across the two different task conditions. Long-term studies should be conducted in the future with a larger PwP population and balanced population groups to further evaluate the performance of the adaptive framework.

## Data Availability

The datasets presented in this article are not readily available because the raw data supporting the conclusion of this article will not be available as per the ethics agreement with the hospital to protect participant confidentiality and privacy.

## Footnotes

1 Note that while participants may have been subjected to different experiment procedures/changes in adaptive framework parameters, the only factor being modeled is the OA/PwP divide when comparing the overall performance. The rationale is further discussed in Section 3.3.2
